# The need for a clinical case definition in test-negative design studies estimating vaccine effectiveness

**DOI:** 10.1038/s41541-023-00716-9

**Published:** 2023-08-12

**Authors:** Sheena G. Sullivan, Arseniy Khvorov, Xiaotong Huang, Can Wang, Kylie E. C. Ainslie, Joshua Nealon, Bingyi Yang, Benjamin J. Cowling, Tim K. Tsang

**Affiliations:** 1 WHO Collaborating Centre for Reference and Research on Influenza, Royal Melbourne Hospital, and Department of Infectious Diseases, University of Melbourne, at the Peter Doherty Institute for Infection and Immunity, Melbourne, VIC Australia; 2 Department of Epidemiology, Fielding School of Public Health, University of California Los Angeles, Los Angeles, CA USA; 3 WHO Collaborating Centre for Infectious Disease Epidemiology and Control, School of Public Health, Li Ka Shing Faculty of Medicine, The University of Hong Kong, Hong Kong Special Administrative Region, China; 4 Centre for Infectious Disease Control, National Institute for Public Health and the Environment, Bilthoven, the Netherlands; 5 Laboratory of Data Discovery for Health Limited, Hong Kong Science and Technology Park, New Territories, Hong Kong Special Administrative Region, China

**Keywords:** Epidemiology, Influenza virus, Epidemiology

## Abstract

Test negative studies have been used extensively for the estimation of COVID-19 vaccine effectiveness (VE). Such studies are able to estimate VE against medically-attended illness under certain assumptions. Selection bias may be present if the probability of participation is associated with vaccination or COVID-19, but this can be mitigated through use of a clinical case definition to screen patients for eligibility, which increases the likelihood that cases and non-cases come from the same source population. We examined the extent to which this type of bias could harm COVID-19 VE through systematic review and simulation. A systematic review of test-negative studies was re-analysed to identify studies ignoring the need for clinical criteria. Studies using a clinical case definition had a lower pooled VE estimate compared with studies that did not. Simulations varied the probability of selection by case and vaccination status. Positive bias away from the null (i.e., inflated VE consistent with the systematic review) was observed when there was a higher proportion of healthy, vaccinated non-cases, which may occur if a dataset contains many results from asymptomatic screening in settings where vaccination coverage is high. We provide an html tool for researchers to explore site-specific sources of selection bias in their own studies. We recommend all groups consider the potential for selection bias in their vaccine effectiveness studies, particularly when using administrative data.

## Introduction

Since the initial roll-out of COVID-19 vaccines, the test-negative design has been frequently applied to enable timely monitoring of COVID-19 vaccine effectiveness (VE)^1^. This design has been extensively used for estimation of influenza VE^2^, for which studies have often leveraged sentinel surveillance systems where patients presenting with a particular clinical case definition are enroled from ambulatory or inpatient medical facilities, regardless of their vaccination status, and tested for the pathogen of interest. Those patients testing positive are identified as cases, while those testing negative are identified as non-cases. VE is estimated from the odds ratio comparing the odds of vaccination among the cases versus non-cases, adjusting for important confouders^3,4^. Here, the term “non-cases” is deliberately used because case status is not known at the time of enrolment, and no sampling frame is used to guide recruitment of cases and non-cases, which differentiates the test-negative design from the traditional case-control study.

The test-negative design has been extensively validated for influenza^4–9^, usually under the scenario described above. We have previously reviewed its application to other pathogens and have cautioned that its suitability needs to be re-examined for each new use^2^. The applicability of the test-negative design for monitoring COVID-19 VE was not examined until after widespread use and several possible weaknesses were highlighted^10^.

**Fig. 1:**
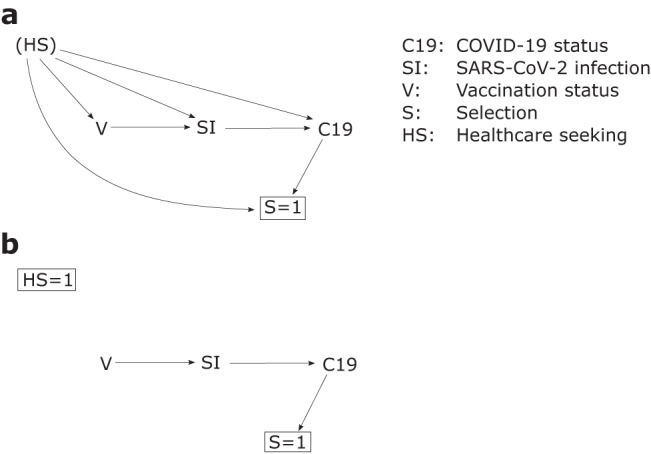
Directed acyclic graph illustrating selection bias in test-negative studies by health-care-seeking behaviour. In (**a**), health-care-seeking behaviour HS confounds the relationship between vaccination V, SARS-CoV-2 infection status SI (e.g., by influencing engagement in risk behaviours), and COVID-19 status C19 (e.g., because of other healthy behaviours that modify disease severity). Only patients who are tested for SARS-CoV-2 are selected into the study *S* = 1. An individual’s health-care-seeking behaviour HS and COVID-19 status C19 influence whether they present for care, are tested and selected into the study *S* = 1, resulting in collider bias. In (**b**), the test-negative design by restricting participants to those who present to sentinel sites and meet particular clinical criteria HS = 1, the collider bias introduced by *S* = 1 is blocked enabling unbiased estimation of the V-C19 effect.

Here, we focus on one key design feature of the test-negative design that has been variously implemented: the restriction of participants to those meeting a clinical case definition. Prior to COVID-19, laboratory tests for confirmation of infection were typically only conducted on people with clinical symptoms. However, given the pre-symptomatic transmission potential of COVID-19 cases, laboratory tests were conducted on many people without symptoms, so some studies using the test-negative design may include participants that would not meet a clinical case definition. Notwithstanding other sources of bias, the use of a clinical case definition is an attempt to ensure that cases and non-cases are derived from the same source population; i.e., patients who would have presented for care with the disease of interest and been enroled as cases had they tested positive for the pathogen of interest. The causal model is depicted in Fig. 1.

Clinical restriction underscores two key features of test-negative studies. First, in this design, VE is not estimated against infection per se, but estimates the vaccine’s effectiveness at preventing medically-attended illness (or hospitalised illness, if enrolment is in hospitals). Second, failure to restrict the population in this way breaks the assumption that cases and non-cases are derived from the same source population^10^. This problem relates to the selection bias that might be induced by differential health seeking between cases and non-cases^6,8,10^. Lewnard et al. explored this problem and noted that in scenarios where healthcare seeking is correlated with vaccination, ignoring it inflates VE estimates^10^.

Studies using health services databases may be at greatest risk of this selection bias. These studies typically use data collected for administrative purposes rather than for the study in question. They may assimilate results on a broad range of individuals tested for a variety of reasons. For example, administrative datasets may include a high proportion of people tested asymptomatically as part of screening programmes, close contacts tested to clear isolation, or the worried well. The pool of negative test results may be over-represented by people whose degree of risk was associated with their vaccination status (e.g., because their workplace requires both asymptomatic screening and vaccination), which can result in a higher proportion of unvaccinated cases leading to higher VE estimates.

## Evidence from a systematic review

To demonstrate the problem, we explored VE estimates extracted as part of a systematic review^1^ of test-negative design studies that estimated VE against medically attended COVID-19 illness and severe disease (hospitalisation, admission to intensive care unit and/or death) for a primary course of vaccination. Full details are provided elsewhere^1^, but briefly, papers were included if the authors described the study as a test-negative design or all participants included in the analysis had been tested for SARS-CoV-2, irrespective of clinical criteria. Data were extracted using a standard data collection form, which included whether or not the study used clinical criteria for enrolment.

**Fig. 2:**
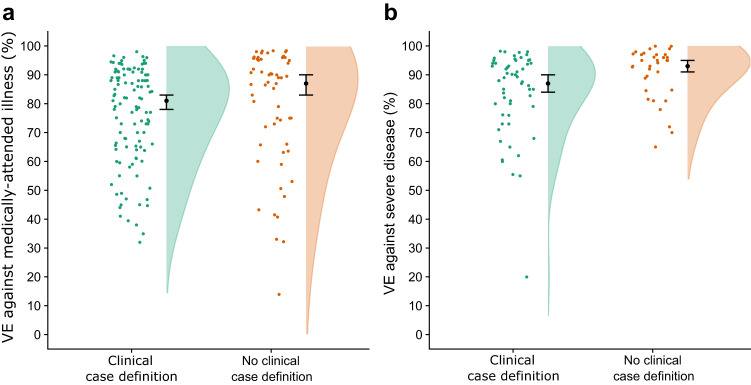
Summary of VE estimates for 66 studies included in systematic review and published between January 2021 and July 2022. **a** shows VE against medically-attended illness, while (**b**) shows VE against severe disease. Points indicate the VE point estimate from each study without confidence intervals. Black points with lines show the pooled estimate from the random-effect meta-analysis with 95% confidence intervals. Shaded area is the violin plot, which is the smoothed density of the VE point estimates.

The search was last updated 11 July 2022 and identified 66 studies that met our inclusion criteria (Supplementary Table 1). Forty-one studies used clinical criteria for enrolment, while 25 did not (Supplementary Tables 1 and 2). Pooled VE was estimated using random effects meta-analysis. VE against medically-attended illness from studies that did not use clinical criteria was higher (VE: 87%; 95% CI: 83%, 90%) than studies that used clinical criteria (VE: 81%; 95% CI: 78%, 83%; Fig. 2), representing a ratio of odds ratios (ROR), 1.44 (95% CI: 1.08, 1.91). VE against severe disease was also higher in studies that did not use clinical criteria (VE: 93%; 95% CI: 91%, 95% versus VE: 87%; 95% CI: 84%, 90%; Fig. 2), corresponding to an ROR of 1.92 (95% CI: 1.30, 2.85). In meta-regression these ratios were recalculated adjusting for whether the study included participants with prior infection, the predominant SARS-CoV-2 circulating variant and the type of vaccine used. These adjustments reduced the RORs to 1.17 (95% CI: 0.95, 1.46) for medically-attended illness and 1.48 (95% CI: 1.08, 2.04) for severe illness, suggesting that clinical criteria may be more important for studies of severe disease.

We note that some studies using administrative data have restricted the study sample to individuals with certain discharge codes to approximate a clinical case definition^11^. However, discharge diagnoses are assigned after testing, so this approach may still fail to achieve exchangeability between cases and non-cases in terms of their clinical indications for testing. Moreover, such an approach is contingent on assuming that testing was not influenced by the patient’s vaccination status. When test-negative studies are run prospectively, participating providers can be reminded to remain impartial about vaccination status when sampling patients.

## Evidence from simulations

We also sought to demonstrate the impact of this form of selection bias using a simple simulation. The associated R script is provided in the Supplementary Information and at https://github.com/khvorov45/casedef. We assumed cases are all people with a positive test result, which includes people infected with SARS-CoV-2 who have symptoms (e.g., identified through symptomatic testing) and people infected with SARS-CoV-2 who do not have symptoms (e.g., identified through asymptomatic screening). Non-cases are all people with a negative test result, some of whom have symptoms and are infected with anything other than SARS-CoV-2, and some of whom have no symptoms and are not infected with SARS-CoV-2 (we will call them “healthy” to differentiate them from people who have an infection). Table 1 shows the default simulation parameters under which the VE estimate from a test-negative study is unbiased.

**Table 1:** Default simulation parameters when VE is unbiased.

Parameter	Values
Vaccine effectiveness^a^	60%
Proportion of healthy included as non-cases^b^	
Vaccinated	0%
Unvaccinated	0%
Proportion of asymptomatic included as cases	
Vaccinated	0%
Unvaccinated	0%
Proportion of SARS-CoV 2 infections that are symptomatic	
Vaccinated	50%
Unvaccinated	50%
Risk of SARS-CoV-2^c^ in unvaccinated	1%
Risk SARS-CoV-2^3^ in vaccinated (discounting the effect of vaccination).	1%
Probability of a symptomatic infection with anything other than SARS-CoV-2	20%
Vaccine coverage	70%

^a^Vaccine effectiveness is against any SARS-CoV-2 infection, regardless of symptoms.

^b^The “proportion of healthy included as non-cases” for the vaccinated means the proportion of the population (who are vaccinated and healthy) that are included into the study as non-cases. When this parameter is 0, no vaccinated healthy person is included into the study (as is the case in true test-negative studies). When this parameter is at 100%, the entire vaccinated, non-case population is comprised of ‘healthy’ people who are uninfected with the target pathogen (as is the case in case-control studies). Similar logic applies to this parameter for the unvaccinated.

^c^The risk of SARS-CoV-2 means probability of true SARS-CoV-2 infection (either symptomatic or asymptomatic) given enrolment into the study.

**Fig. 3:**
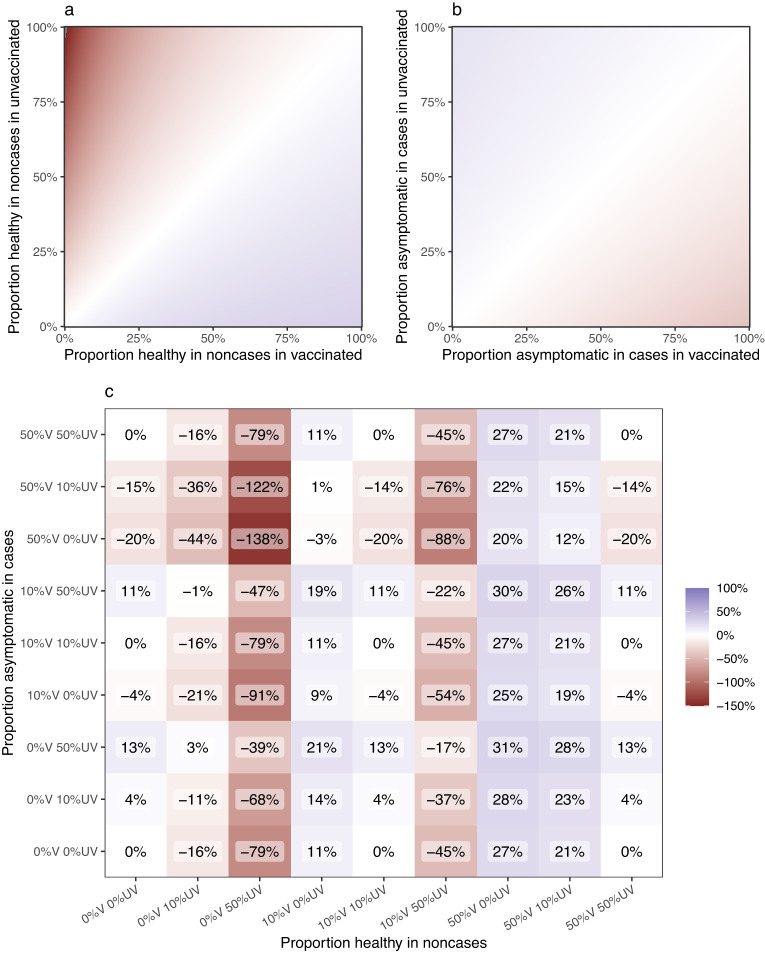
Expected bias in VE estimates under various assumptions about the clinical case definition. Expected bias (estimated VE minus true VE) is shown at different values of proportion of asymptomatic (healthy) people who are part of the study as non-cases and proportion of asymptomatically people who are included as cases (proportion is the same for the vaccinated and the unvaccinated). It show the bias when the proportion asymptomatic is differential by vaccination status in non-cases (**a**) and cases (**b**). The non-differential case is also shown along the diagonal in (**a**) and (**b**) and while non-zero is negligible and not visible on the plot. Note that for (**b**) this is because the proportion of asymptomatic infections among all infections is the same for the vaccinated and the unvaccinated in the simulation under the default parameter set. **c** It shows selected values exploring the bias at different asymptomatic proportions by both vaccination and case status. Axis labels are understood as follows: “25%V 75%UV” indicates that for the vaccinated the proportion asymptomatic is set to 25%, for the unvaccinated it is set to 75%. For all plots, the percent bias indicates the difference in VE estimate compared with the default value of 60%; e.g., a value of −17% means the estimated value is VE = 47%. All parameters other than the ones in the X and Y axes are set to their default values as per Table 1.

We first explored the scenario where the asymptomatic proportion was allowed to vary by case status but did not vary by vaccination status. The bias in this situation is negligible (diagonals in Fig. 3a, b).

Next, we examined the situation where some proportion of non-cases are healthy and would not be included if using a clinical case definition. This scenario might occur if the dataset includes people from workplaces that conduct asymptomatic screening. Figure 3a shows the effect of this bias. If those same workplaces also require vaccination, then the proportion of healthy vaccinated non-cases may be greater than the proportion of healthy unvaccinated non-cases. In this scenario, the expected VE estimate is biased positively away from the null (i.e., bottom right half of Fig. 3a; VE is overestimated). When the proportion of healthy individuals is lower among the vaccinated compared with unvaccinated non-cases, which might occur if eligibility for travel or entertainment entrance is contingent on testing for the unvaccinated, VE is biased towards the null and can even be negative (i.e., top left half of Fig. 3a; VE is underestimated). This bias is negligible at low disease prevalence even in the extreme case of both proportions being 100% (this would be equivalent to a standard case-control study).

The converse scenario showing bias that occurs when the asymptomatic proportion among the cases is varied is shown in Fig. 3b. When vaccination reduces symptoms severity^11^, and the proportion of asymptomatic cases is higher among the vaccinated, the estimate is biased towards the null (i.e., bottom right half of Fig. 3b; VE is underestimated). This might occur if the dataset includes people working or resident in settings where vaccination is high (e.g., aged care) and testing identifies a high proportion of asymptomatic cases through screening during an outbreak. Note, however, that the scenarios in Fig. 3b result in less bias than those depicted in Fig. 3a.

If the asymptomatic proportions among cases and non-cases are not the same for the vaccinated and the unvaccinated, a compounding effect is observed (Fig. 3c). For example, if the proportion asymptomatic in cases is greater in the vaccinated, we know from Fig. 3b the bias will be negative. If the proportion healthy in non-cases is greater in the unvaccinated, we know from Fig. 3a the bias will be negative. When both are true, the bias becomes more negative and pulls estimates further from their true value. In some scenarios, the bias may cancel out, such as when the proportion asymptomatic in cases is greater in the vaccinated, and proportion healthy in non-cases is greater in the vaccinated. To realise the inflated VE seen in the systematic review, the most likely scenario is one where the healthy proportion among vaccinated non-cases is higher than among unvaccinated non-cases (i.e., columns marked 50%V 0%UV or 50%V 10%UV), irrespective of the asymptomatic proportion among the cases. However, there are numerous possible scenarios and the degree of bias will change under different default parameter values. Further combinations of parameter values can be explored using an html tool available at https://github.com/khvorov45/casedef.

## Conclusions

Rapid VE estimation, especially estimation that leverages administrative data and can therefore be done less expensively than studies which follow a sampling framework, is an attractive option. However, research groups and policy makers need to understand the pitfalls of this approach.

The application of a clinical case definition in test-negative studies provides some reassurance that the non-case group reflects the source population of the cases^12^. While this requirement increases the likelihood that the non-cases have a similar risk of exposure to the SARS-CoV-2 virus, it does not guarantee it. Some non-cases may still fail to meet the exposure necessity assumption^12^; i.e., some non-cases may not, in fact, have been exposed to the virus and were therefore never at risk of COVID-19 illness. Moreover, the use of clinical criteria seeks to address internal validity; generalisability is limited to the healthcare seeking population^13^. In some special cases, it may be possible to estimate VE in the whole population; for example, when participants are recruited through point-prevalence surveys^14^ or in studies that limit participants to close contacts of a case such as household transmission studies^15^. However, those approaches may still suffer from participation bias^13^.

Salvaging internal validity, at a minimum, is important for public health decision making. In VE studies, generalising to the healthcare-seeking population may be satisfactory since it is the burden on our health systems we wish to mitigate with vaccination. Where selection processes fail to ensure the study sample represents the source population, various methods exist to correct the resultant selection bias, but may require additional information unavailable to the researcher^16–19^. We recommend that all research groups perform an assessment of the degree to which VE is biased under selection scenarios relevant to their setting. The tool we have provided can help with this assessment.

When conducted with a clinical case definition in mind, test-negative studies may be able to provide valid estimates of VE against a specific syndrome of medically-attended disease. When the indications for testing are ignored, the resulting VE is unbiased only when the asymptomatic proportions included into cases and non-cases are the same for the vaccinated and the unvaccinated, which is rare. It is otherwise unclear what the VE estimate represents, but it is unlikely to be a measure of VE against infection, nor medically-attended illness, and is instead some hybrid, the public health implications of which are unclear (and possibly unhelpful). If the goal is to estimate VE against infection, not disease, the test-negative design is not the best design choice, and those choosing it need to acknowledge fully its limitations. The tool we have provided in the supplementary information can help researchers assess the potential for bias under scenarios most plausible for their population.

### Reporting summary

Further information on research design is available in the Nature Research Reporting Summary linked to this article.

### Supplementary information

Supplementary Information

Reporting Summary

## Data Availability

Papers included in systematic review are listed in the appendix. Any further data extracted from reviewed articles can be provided upon request to Tim K. Tsang timkltsang@gmail.com.

## Code availability

R and html scripts used in simulations are available https://github.com/khvorov45/casedef. In addition, the R scripts for the simulations are provided with the Supplementary Information. R scripts used for meta-analysis and meta-regression can be provided upon request to timkltsang@gmail.com.
