# Supplementary material for: The need for a clinical case definition in test-negative design studies estimating vaccine effectiveness
Source: NPJ Vaccines. 2023 Aug 12;8:118. doi: 10.1038/s41541-023-00716-9 (PMC10423262; doi:10.1038/s41541-023-00716-9)
Supplement: Supplementary file 1 — Supplementary Information [file 41541_2023_716_MOESM1_ESM.pdf]

The need for a clinical case definition in test-negative design studies estimating vaccine effectiveness

Supplementary material

Sheena G. Sullivan, Arseniy Khvorov, Xiaotong Huang, Can Wang, Kylie E.C. Ainslie, Joshua Nealon, Bingyi Yang, Benjamin J. Cowling, Tim K. Tsang

July 18, 2023

Contents

1 R script used for simulations 2

2 Supplementary Tables: Studies included in systematic review 7

1 R script used for simulations

see also https://github.com/khvorov45/casedef

```
# Author: Arseniy Khvorov
# Date: 6 June 2023
# Purpose: Simulations exploring the influence of the asymptomatic proportion
# among cases and noncases in test negative studies of COVID-19 VE

library(tidyverse)

expected_ve <- function(
    target_infection_unvac = 0.01,
    target_infection_vac_veo = 0.01,
    ve = 0.6,
    target_symptoms_unvac = 0.5,
    target_symptoms_vac = 0.5,
    nontarget_symptomatic_infection = 0.2,
    asympt_in_cases_vac = 0,
    asympt_in_cases_unvac = 0,
    healthy_in_noncases_unvac = 0,
    healthy_in_noncases_vac = 0,
    vaccine_coverage = 0.7,
    name = "name"
) {
    target_infection_vac <- target_infection_vac_veo * (1 - ve)

    healthy_vac <- 1 - target_infection_vac - nontarget_symptomatic_infection
    healthy_unvac <- 1 - target_infection_unvac - nontarget_symptomatic_infection

    asympt_vac <- 1 - target_symptoms_vac
    asympt_unvac <- 1 - target_symptoms_unvac

    vaccinated_cases <- vaccine_coverage * target_infection_vac *
      (target_symptoms_vac + asympt_vac * asympt_in_cases_vac)
    vaccinated_noncases <- vaccine_coverage *
      (nontarget_symptomatic_infection + healthy_vac * healthy_in_noncases_vac)

    unvac_cases <- (1 - vaccine_coverage) * target_infection_unvac *
      (target_symptoms_unvac + asympt_unvac * asympt_in_cases_unvac)
    unvac_noncases <- (1 - vaccine_coverage) *
      (nontarget_symptomatic_infection + healthy_unvac *
         healthy_in_noncases_unvac)

    odds_vaccination_cases <- vaccinated_cases / unvac_cases
    odds_vaccination_noncases <- vaccinated_noncases / unvac_noncases

    or <- odds_vaccination_cases / (odds_vaccination_noncases)
    est_ve <- 1 - or

    tibble(
        target_infection_unvac,
        target_infection_vac_veo,
        true_ve = ve,
        target_symptoms_unvac,
        target_symptoms_vac,
        nontarget_symptomatic_infection,
        asympt_in_cases_vac,
        asympt_in_cases_unvac,
        healthy_in_noncases_unvac,
        healthy_in_noncases_vac,
```

```
        vaccine_coverage ,
        name,
        target_infection_vac ,
        healthy_vac ,
        healthy_unvac ,
        asympt_vac,
        asympt_unvac,
        vaccinated_cases ,
        vaccinated_noncases ,
        unvac_cases ,
        unvac_noncases ,
        odds_vaccination_cases ,
        odds_vaccination_noncases ,
        or ,
        est_ve ,
        addbias = est_ve - true_ve,
        mulbias = 1 - (1 - est_ve) / (1 - true_ve)
    )
}

npoints <- 300

args_asympt_in_cases_and_healthy_in_noncases_by_vac_status <- expand.grid(
    asympt_in_cases_vac = c(0, 0.1, 0.5),
    asympt_in_cases_unvac = c(0, 0.1, 0.5),
    healthy_in_noncases_vac = c(0, 0.1, 0.5),
    healthy_in_noncases_unvac = c(0, 0.1, 0.5)
)

res <- bind_rows(
    expected_ve(
        healthy_in_noncases_unvac = rep(seq(0, 1, length.out = npoints),
                                        each = npoints),
        healthy_in_noncases_vac = rep(seq(0, 1, length.out = npoints),
                                      times = npoints),
        name = "healthy_in_noncases"
    ),
    expected_ve(
        target_infection_vac_veo = rep(seq(0.005, 0.02, length.out = npoints),
                                       each = npoints),
        target_infection_unvac = rep(seq(0.005, 0.02, length.out = npoints),
                                     times = npoints),
        name = "target_infection"
    ),
    expected_ve(
        target_symptoms_vac = rep(seq(0.2, 0.8, length.out = npoints),
                                  each = npoints),
        target_symptoms_unvac = rep(seq(0.2, 0.8, length.out = npoints),
                                    times = npoints),
        name = "target_symptoms"
    ),
    expected_ve(
        asympt_in_cases_vac = rep(seq(0, 1, length.out = npoints),
                                  each = npoints),
        asympt_in_cases_unvac = rep(seq(0, 1, length.out = npoints),
                                    times = npoints),
        name = "asympt_in_cases"
    ),
    expected_ve(
        asympt_in_cases_vac = rep(seq(0, 1, length.out = npoints),
```

```
                                    each = npoints),
        asympt_in_cases_unvac = rep(seq(0, 1, length.out = npoints),
                                    each = npoints),
        healthy_in_noncases_vac = rep(seq(0, 1, length.out = npoints),
                                      times = npoints),
        healthy_in_noncases_unvac = rep(seq(0, 1, length.out = npoints),
                                        times = npoints),
        name = "asympt_in_cases_and_healthy_in_noncases"
    ),

    expected_ve(
        asympt_in_cases_vac =
          args_asympt_in_cases_and_healthy_in_noncases_by_vac_status$asympt_in_cases_v
        asympt_in_cases_unvac =
          args_asympt_in_cases_and_healthy_in_noncases_by_vac_status$asympt_in_cases_u
        healthy_in_noncases_vac =
          args_asympt_in_cases_and_healthy_in_noncases_by_vac_status$healthy_in_noncas
        healthy_in_noncases_unvac =
          args_asympt_in_cases_and_healthy_in_noncases_by_vac_status$healthy_in_noncas
        name = "asympt_in_cases_and_healthy_in_noncases_by_vac_status"
    )
)

write_csv(res, "res.csv")

genall <- function(biasname) {

    ggsave2 <- function(name, plot, ...) {
        ggsave(paste0(name, "_", biasname, ".pdf"), plot, units = "cm", ...)
        ggsave(paste0(name, "_", biasname, ".png"), plot, units = "cm", ...)
    }

    plot_comp <- list(
        theme_bw(),
        scale_fill_gradient2("Bias", labels = scales::percent_format(),
                             limits = c(-1.5, 1)),
        scale_color_gradient2("Bias", labels = scales::percent_format(),
                              limits = c(-1.5, 1)),
        geom_tile(aes(fill = !!sym(biasname), color = !!sym(biasname)))
    )

    plot_healthy_in_noncases <- res %>%
        filter(name == "healthy_in_noncases") %>%
        ggplot(aes(healthy_in_noncases_vac, healthy_in_noncases_unvac)) +
        plot_comp +
        scale_x_continuous("Proportion healthy in noncases in vaccinated",
                           expand = expansion(0, 0),
                           labels = scales::percent_format()) +
        scale_y_continuous("Proportion healthy in noncases in unvaccinated",
                           expand = expansion(0, 0),
                           labels = scales::percent_format())

    width <- 13
    height <- 10

    ggsave2("healthy_in_noncases", plot_healthy_in_noncases,
            width = width, height = height)

    plot_target_infection <- res %>%
        filter(name == "target_infection") %>%
```

```
    ggplot(aes(target_infection_vac_veo, target_infection_unvac)) +
    plot_comp +
    scale_x_continuous("Risk of target infection in vaccinated",
                       expand = expansion(0, 0),
                       labels = scales::percent_format()) +
    scale_y_continuous("Risk of target infection in unvaccinated",
                       expand = expansion(0, 0),
                       labels = scales::percent_format())

ggsave2("target_infection", plot_target_infection,
        width = width, height = height)

plot_target_symptoms <- res %>%
    filter(name == "target_symptoms") %>%
    ggplot(aes(target_symptoms_vac, target_symptoms_unvac)) +
    plot_comp +
    scale_x_continuous("Proportion of symptomatic infections in vaccinated",
                       expand = expansion(0, 0),
                       labels = scales::percent_format()) +
    scale_y_continuous("Proportion of symptomatic infections in unvaccinated",
                       expand = expansion(0, 0),
                       labels = scales::percent_format())

ggsave2("target_symptoms", plot_target_symptoms,
        width = width, height = height)

plot_asympt_in_cases <- res %>%
    filter(name == "asympt_in_cases") %>%
    ggplot(aes(asympt_in_cases_vac, asympt_in_cases_unvac)) +
    plot_comp +
    scale_x_continuous("Proportion asymptomatic in cases in vaccinated",
                       expand = expansion(0, 0),
                       labels = scales::percent_format()) +
    scale_y_continuous("Proportion asymptomatic in cases in unvaccinated",
                       expand = expansion(0, 0),
                       labels = scales::percent_format())

ggsave2("asympt_in_cases", plot_asympt_in_cases,
        width = width, height = height)

plot_asympt_in_cases_and_healthy_in_noncases <- res %>%
    filter(name == "asympt_in_cases_and_healthy_in_noncases") %>%
    ggplot(aes(asympt_in_cases_vac, healthy_in_noncases_vac)) +
    theme_bw() +
    scale_fill_gradient2("Bias", labels = scales::percent_format()) +
    scale_color_gradient2("Bias", labels = scales::percent_format()) +
    geom_tile(aes(fill = !!sym(biasname), color = !!sym(biasname))) +
    scale_x_continuous("Proportion asymptomatic in cases",
                       expand = expansion(0, 0),
                       labels = scales::percent_format()) +
    scale_y_continuous("Proportion healthy in noncases",
                       expand = expansion(0, 0),
                       labels = scales::percent_format())

ggsave2("asympt_in_cases_and_healthy_in_noncases",
        plot_asympt_in_cases_and_healthy_in_noncases,
        width = width, height = height)

plot_asympt_in_cases_and_healthy_in_noncases_by_vac_status <- res %>%
  filter(name == "asympt_in_cases_and_healthy_in_noncases_by_vac_status") %>%
```

```
      mutate(asympt_in_cases = glue::glue("{asympt_in_cases_vac * 100}%V
                                          {asympt_in_cases_unvac * 100}%UV") %>%
               fct_reorder(asympt_in_cases_unvac) %>%
               fct_reorder(asympt_in_cases_vac),
             healthy_in_noncases =
               glue::glue("{healthy_in_noncases_vac * 100}%V
                          {healthy_in_noncases_unvac * 100}%UV") %>%
            fct_reorder(healthy_in_noncases_unvac) %>%
              fct_reorder(healthy_in_noncases_vac),) %>%
        select(!!sym(biasname), asympt_in_cases, healthy_in_noncases) %>%
      ggplot(aes(healthy_in_noncases, asympt_in_cases)) + plot_comp +
        geom_label(aes(label = round(!!sym(biasname) * 100) %>% paste0("%")),
                   alpha = 0.5, label.size = 0) +
        theme(axis.text.x = element_text(angle = 30, hjust = 1)) +
        scale_x_discrete("Proportion healthy in noncases",
                         expand = expansion(0, 0)) +
        scale_y_discrete("Proportion asymptomatic in cases",
                         expand = expansion(0, 0))

    ggsave2("asympt_in_cases_and_healthy_in_noncases_by_vac_status",
            plot_asympt_in_cases_and_healthy_in_noncases_by_vac_status,
            width = width * 1.5, height = height * 1.5)

    combined <- ggpubr::ggarrange(
        ggpubr::ggarrange(
            plot_healthy_in_noncases +
                theme(legend.position = "none",
                      plot.margin = margin(10, 15, 10, 10)) +
                ggtitle("a"),
            plot_asympt_in_cases +
                theme(legend.position = "none",
                      plot.margin = margin(10, 15, 10, 10)) +
                ggtitle("b")
        ),
        plot_asympt_in_cases_and_healthy_in_noncases_by_vac_status +
                ggtitle("c") +
                theme(legend.title = element_blank()),
        ncol = 1,
        heights = c(1, 1.5)
    )

    ggsave2("combined", combined, width = 20, height = 25)
}

genall("addbias")
genall("mulbias")
```

2 Supplementary Tables: Studies included in systematic review

Supplementary Table 1: Summary of 66 studies included in the systematic review and meta-analysis. Methods described in (1).

**Author (year)**	**Age group**	**Study period**	**Location**	**Vaccine type**	**Circulating viruses**	**Endpoint type**	**Recruitment criteria**	**Adjustments in analysis**
Amirthalingam (2021) (2)	≤50 years	2020-10-26 to 2021-06-18	UK	mRNA & Adenovirus vector vaccines	Alpha	MAI	Clinical	Week of onset, 5-year age bands, gender, NHS region, index of multiple deprivation (quintiles), ethnicity, health/social care worker, care home resident
Kim (2021) (3)	≥16 years	2021-02-01 to 2021-05-28	US	mRNA	Alpha	MAI	Clinical	Study site, age in years, enrolment period, race and ethnicity, and contact with a SARS-CoV-2–positive person
Maeda (2022) (4)	≥16 years	2021-07-01 to 2021-09-30	Japan	mRNA	Delta	MAI	Clinical	Age, sex, presence of underlying medical conditions, calendar week, history of contact with COVID-19 patients, and medical institution
Mallow (2022) (5)	18 years	2021-01-01 to 2021-08-25	US	mRNA	Mix	MAI	Clinical	Age, gender, race, insurance status, imputed body mass index [BMI], vaccine type, week of presentation
Ranzani2 (2022) (6)	all ages	2021-01-17 to 2021-11-27	Brazil	Adenovirus vector vaccines	Gamma & delta	MAI	Clinical	Age, sex, cardiovascular disease, respiratory disease, obesity, diabetes mellitus, immunosuppressed status, liver disease, occupation, region of residence, self-reported race, reason of testing, and day of the year of RT-qPCR testing symptomatic and asymptomatic
Sheikh (2021) (7)	all ages	2021-04-01 to 2021-06-06	UK	mRNA & Adenovirus vector vaccines	Alpha & delta	MAI	Clinical	Age, number of prior COVID tests, date and factors for sex and deprivation
Tabak (2021) (8)	≥18 years	2021-05-01 to 2021-08-07	US	mRNA & Adenovirus vector vaccines	Delta	MAI	Clinical	Age, region, and calendar month of test

Supplementary Table 1: Summary of 66 studies included in the systematic review and meta-analysis. Methods described in (1).

Author (year)	Age group	Study period	Location	Vaccine type	Circulating viruses	Endpoint type	Recruitment criteria	Adjustments in analysis
Andrews2 (2022) (9)	≥16 years	2020-12-08 to 2021-10-01	UK	mRNA & Adenovirus vector vaccines	Alpha & delta	MAI & severe	Clinical	Age, sex, index of multiple deprivation (a measure of socioeconomic status), race or ethnic group, care home residence status (for analyses including persons ?65 years of age), geographic region, period (calendar week), health and social care worker status (for analyses involving persons <65 years of age), and status of being in a clinical risk group (available only for persons <65 years of age) or a clinically extremely vulnerable group (any age)
Cerqueira-Silva (2022) (10)	≥18 years	2021-01-18 to 2021-11-11	Brazil	Inactivated virus vaccines	Gamma & delta	MAI & severe	Clinical	Age, sex, temporal trends, state of residence, previous MAI, pregnancy, postpartum period and comorbidities
Drawz (2022) (11)	≥19 years	2021-08-29 to 2021-11-27	US	mRNA	Delta	MAI & severe	Clinical	Demographic groups and those with high risk conditions for COVID-19 disease with at least 6 events and more than 25,000 person-weeks at risk.
Hitchings (2021) (12)	≥60 years	2021-01-17 to 2021-07-02	Brazil	Adenovirus vector vaccines	Gamma	MAI & severe	Clinical	The number of reported comorbidities, previous positive SARS-CoV-2 RT-PCR or antigen test, and age
Nadeem (2022) (13)	≥60 years	2021-05-05to2021-07-31	Pakistan	mRNA	Delta	MAI & severe	Clinical	NA
Ranzani1 (2021) (14)	≥70 years	2021-01-17 to 2021-04-29	Brazil	Inactivated virus vaccines	Gamma	MAI & severe	Clinical	Age and number of comorbidities
Rearte (2022) (15)	≥60 years	2021-01-31 to 2021-09-14	Argentina	Adenovirus vector vaccines & Inactivated virus vaccines	Gamma & lambda & alpha	MAI & severe	Clinical	Epidemiological week, age, sex, history of COVID-19, and district

Supplementary Table 1: Summary of 66 studies included in the systematic review and meta-analysis. Methods described in (1).

**Author (year)**	**Age group**	**Study period**	**Location**	**Vaccine type**	**Circulating viruses**	**Endpoint type**	**Recruitment criteria**	**Adjustments in analysis**
Thompson (2021) (16)	≥50 years	2021-01-01 to 2021-06-22	US	mRNA & Adenovirus vector vaccines	Mix	MAI & severe	Clinical	Age, geographic region, calendar time, and local virus circulation
Embi (2022) (17)	≥18 years	2021-01-17 to 2021-09-05	US	mRNA	Delta	Severe	Clinical	Age, geographic region, calendar time, and local virus circulation and weighted for inverse propensity to be vaccinated or unvaccinated
Lauring (2022) (18)	≥18 years	2021-03-11 to 2022-01-14	US	mRNA	Alpha & delta & omicron	Severe	Clinical	Number of comorbidities, smoking status, living in a long term care facility before hospital admission, and working in a healthcare setting
Ngyen (2022) (19)	all ages	2020-12-23 to 2021-06-15	France	mRNA	Mix	Severe	Clinical	Time, age, and stratified on centers
Niessen (2022) (20)	≥18 years	2021-03-01to2021-06-26	Netherlands	Adenovirus vector vaccines	Alpha	Severe	Clinical	Age group and week of symptom onset
Price (2022) (21)	12-18 years	2021-07-01 to 2022-02-17	US	mRNA	Delta & omicron	Severe	Clinical	Sex, age, race, region, calendar time
Self (2021) (22)	≥18 years	2021-03-11 to 2021-08-15	US	mRNA & Adenovirus vector vaccines	Alpha & delta	Severe	Clinical	Admission date, geographic region, age, sex, and race and Hispanic ethnicity
Tartof (2022) (23)	≥18 years	2021-12-01to2022-02-06	US	mRNA	Delta & omicron	Severe	Clinical	Age, sex, race/ethnicity, BMI, Charlson comorbidity index, prior SARS-CoV-2 MAI, prior influenza vaccination, prior pneumococcal vaccination
Tenforde (2021) (24)	≥65 years	2021-01-01 to 2021-03-26	US	mRNA	Wild type & alpha	Severe	Clinical	Region, calendar month, age, sex, and race and ethnicity
Tenforde2 (2022) (25)	≥18 years	2021-03-11to2021-05-05	US	mRNA	Alpha	Severe	Clinical	Calendar time in biweekly intervals, US Department of Health and Human Services region, age in years, sex, and race and ethnicity

Supplementary Table 1: Summary of 66 studies included in the systematic review and meta-analysis. Methods described in (1).

**Author (year)**	**Age group**	**Study period**	**Location**	**Vaccine type**	**Circulating viruses**	**Endpoint type**	**Recruitment criteria**	**Adjustments in analysis**
Tenforde3 (2022) (26)	≥18 years	2021-03-11to2021-12-15	US	mRNA	Delta	Severe	Clinical	Calendar date of admission (in biweekly intervals), age, sex, and race and ethnicity, presence of underlying chronic conditions, immunocompromised status, and US Health and Human Services region of the admitting hospital
Andrews1 (2022) (27)	≥18 years	2021-11-27 to 2022-01-12	UK	mRNA & Adenovirus vector vaccines	Delta & omicron	Symptomatic	Clinical	Age (18 to 89 years in 5-year bands, then everyone ?90 years), sex, index of multiple deprivation (quintile), race or ethnic group, history of foreign travel, geographic region, period (day of test), health and social care worker status, clinical risk group status, status of being in a clinically extremely vulnerable group, and previously testing posit
Bernal1 (2021) (28)	≥16 years	2020-10-26 to 2021-05-16	UK	mRNA & Adenovirus vector vaccines	Alpha & delta	Symptomatic	Clinical	Period (calendar week), travel history, race or ethnic group, sex, age, index of multiple deprivation, clinically extremely vulnerable group, region, history of positive test, health or social care worker, and care home residence
Bernal2 (2021) (29)	≥70 years & ≥80 years	2020-12-08 to 2021-02-19	UK	mRNA & Adenovirus vector vaccines	Alpha	Symptomatic	Clinical	Age, period, sex, region, ethnicity, care home, and index of multiple deprivation fifth
Britton (2022) (30)	≥20 years	2021-03-13 to 2021-10-17	US	mRNA	Delta & mix	Symptomatic	Clinical	Age group, race, ethnicity, sex, testing site state, testing site census tract SVI, and calendar date
Chung2 (2022) (31)	≥12 years	2021-02-01 to 2021-09-30	US	mRNA	Mix	Symptomatic	Clinical	Study site, age in years (continuous), enrolment period, and self-reported race and ethnicity

Supplementary Table 1: Summary of 66 studies included in the systematic review and meta-analysis. Methods described in (1).

**Author (year)**	**Age group**	**Study period**	**Location**	**Vaccine type**	**Circulating viruses**	**Endpoint type**	**Recruitment criteria**	**Adjustments in analysis**
Fleming-Dutra (2022) (32)	5-15 years	2021-12-26to2022-02-21	US	mRNA	Omicron	Symptomatic	Clinical	Calendar day of test (continuous variable), race, ethnicity, sex, testing site region, and testing site census tract Social Vulnerability Index
Powell (2022) (33)	12-15 years	2021-09-13to2022-01-12	UK	mRNA	Delta & omicron	Symptomatic	Clinical	Age, sex, index of multiple deprivation (quintile), ethnic group, geographic region (NHS region), period (calendar week of onset), clinical risk group status (a separate flag for those aged over and under 16), clinically extremely vulnerable (if aged 16 and above) and previous positivity
Suarez Castillo (2022) (34)	≥18 years	2021-12-13to2022-01-13	France	mRNA	Omicron/delta	Symptomatic	Clinical	Age, sex, residence, week of testing and presence of a comorbidity qualifying for prioritisation in the vaccination campaign according to the recommendations of the National Health Authority
Whitaker (2022) (35)	≥16 years	2021-05-16to2021-12-07	UK	mRNA	Alpha	Symptomatic	Clinical	Week-NHS region interaction, 5-yr age group, sex, ethnicity, IMD quintile, GP record of prior COVID-19, large household, GP consultation quartile, chapter count, shielding recommendation, overall PRIMIS risk group status (overall only) and latest smoking status
Andrews3 (2022) (36)	≥18 years	2021-09-13to2021-12-05	UK	mRNA & Adenovirus vector vaccines	Delta	Symptomatic & severe	Clinical	Age (5-year bands), sex, index of multiple deprivation (quintile), ethnic group, care-home residence status, geographic region (NHS region), period (calendar week of onset), health and social care worker status, clinical risk group status, clinically extremely vulnerable, severely immunosuppressed and previously testing positive

Supplementary Table 1: Summary of 66 studies included in the systematic review and meta-analysis. Methods described in (1).

Author (year)	Age group	Study period	Location	Vaccine type	Circulating viruses	Endpoint type	Recruitment criteria	Adjustments in analysis
Chung1 (2021) (37)	≥16 years	2020-12-14 to 2021-04-19	Canada	mRNA	Wild type & alpha & beta	Symptomatic & severe	Clinical	Age, sex, public health unit region, biweekly period of test, number of SARS-CoV-2 tests in the 3 months prior to 14 December 2020, presence of any comorbidity that increase the risk of severe COVID-19, receipt of influenza vaccination in current or prior influenza season, and neighbourhood-level household income, persons per dwelling, proportion of persons employed as non-health essential workers, and self-identified visible minority quintiles
Ferdinands (2022) (38)	≥18 years	2021-08-26 to 2022-01-22	US	mRNA	Delta & omicron	Symptomatic & severe	Clinical	Age, local virus circulation, propensity to be vaccinated, and other factors
Grannis (2021) (39)	≥18 years	2021-06-03 to 2021-08-04	US	mRNA & Adenovirus vector vaccines	Delta	Symptomatic & severe	Clinical	Age, geographic region, calendar time, and virus circulation, and weighted for inverse propensity to be vaccinated or unvaccinated
Kirsebom (2022) (40)	≥18 years	2022-01-17to2022-03-31	UK	mRNA	Omicron	Symptomatic & severe	Clinical	Age, sex, index of multiple deprivation (quintile), ethnic group, history of travel, geographic region (NHS region), period (week of test), health and social care worker status, clinical risk group status, clinically extremely vulnerable, and previously testing positive

Supplementary Table 1: Summary of 66 studies included in the systematic review and meta-analysis. Methods described in (1).

**Author (year)**	**Age group**	**Study period**	**Location**	**Vaccine type**	**Circulating viruses**	**Endpoint type**	**Recruitment criteria**	**Adjustments in analysis**
Nasreen (2022) (41)	all ages	2020-12-14 to 2021-08-03	Canada	mRNA & Adenovirus vector vaccines	Alpha & beta & gamma & beta or gamma & delta & non-VOC	Symptomatic & severe	Clinical	Age, sex, public health unit region, period of test, number of SARS-CoV-2 tests in the 3 months before 14 December 2020, presence of any comorbidity that increase the risk of severe COVID-19, receipt of 2019/2020 and/or 2020/2021 influenza vaccination, and Census dissemination area-level quintiles of household income, proportion of persons employed as non-health essential workers, persons per dwelling, and proportion of self-identified visible minorities
Suarez (2022) (42)	≥50 years	2021-01-01to2021-12-12	France	mRNA	Ancestral strains & alpha & beta/gamma & delta	Symptomatic & severe	Clinical	Age (ten-year age brackets), sex, area of residence, week of testing and presence or absence of a comorbidity qualifying for prioritization in the vaccination campaign
Bruxvoort (2021) (43)	≥18 years	2021-03-01 to 2021-07-27	US	mRNA	Alpha & delta & epsilon & gamma & lota & mu & beta & eta & kappa & other variants	MAI	Non-clinical	BMI, smoking, Charlson comorbidity score, frailty index, lung disease, liver disease, kidney disease, immunocompromised status, pregnancy, heart disease, history of COVID-19 MAI, number of outpatient and virtual visits, number of ED visits, number of hospitalizations, preventive care, Medicaid, medical centre area, KPSC physician/employee status, month of specimen collection, specimen type
Butt (2021) (44)	all ages	2020-12-15 to 2021-03-04	US	mRNA	Wild type & alpha	MAI	Non-clinical	Age, sex, race, body mass index, Charlson Comorbidity Index score, and geographic location

Supplementary Table 1: Summary of 66 studies included in the systematic review and meta-analysis. Methods described in (1).

**Author (year)**	**Age group**	**Study period**	**Location**	**Vaccine type**	**Circulating viruses**	**Endpoint type**	**Recruitment criteria**	**Adjustments in analysis**
Husin (2022) (45)	12-17 years	2021-09-01to2021-12-31	Malaysia	mRNA	Delta	MAI	Non-clinical	Age, sex, states of residence, strata (urban/rural), school types, and number of baseline (before September 1, 2021) tests
Pardo-Seco (2022) (46)	≥18 years	2020-12-27 to 2021-03-18	Spain	mRNA	Alpha	MAI	Non-clinical	Sex, age and time period between SARS-CoV-2 test and the start of study
Sritipsukho (2022) (47)	≥18 years	2021-07-25 to 2021-10-23	Thailand	Adenovirus vector vaccines & Inactivated virus vaccines	Delta	MAI	Non-clinical	Healthcare workers, comorbidities, age, educational level, and sex
Abu-Raddad1 (2022) (48)	all ages	2021-01-01 to 2021-12-05	Qatar	mRNA	Beta & delta	MAI & severe	Non-clinical	Prior MAI, healthcare worker status
Abu-Raddad2 (2021) (49)	all ages	2021-02-01 to 2021-03-31	Qatar	mRNA	Alpha & Beta & other variants	MAI & severe	Non-clinical	Sex, age, nationality, PCR test date
Andrejko (2021) (50)	≥18 years	2021-02-24 to 2021-04-29	US	mRNA	Epsilon & alpha	MAI & severe	Non-clinical	Age, region, sex, income, and race predicted the likelihood an individual was vaccine hesitant
Chemaitelly1 (2021) (51)	all ages	2021-01-01 to 2021-09-05	Qatar	mRNA	Alpha & beta & delta	MAI & severe	Non-clinical	Prior MAI and healthcare worker status
Chemaitelly2 (2021) (52)	all ages	2020-12-28 to 2021-05-10	Qatar	mRNA	Alpha & beta & mix	MAI & severe	Non-clinical	Sex, age, nationality, reason for PCR testing and calendar week

Supplementary Table 1: Summary of 66 studies included in the systematic review and meta-analysis. Methods described in (1).

**Author (year)**	**Age group**	**Study period**	**Location**	**Vaccine type**	**Circulating viruses**	**Endpoint type**	**Recruitment criteria**	**Adjustments in analysis**
Corrao (2022) (53)	all ages	2020-12-27 to 2021-07-16	Italy	mRNA	Alpha & delta	MAI & severe	Non-clinical	The number of previous contacts with the Regional Health Service, use of corticosteroids, drugs for chronic pain, oral anticoagulant agents and insulin, and the presence of anaemias, chronic respiratory disease, dyslipidaemia, depression, hypertension, coronary and peripheral vascular disease, hypothyroidism, epilepsy and recurrent seizures, psychosis, diabetes without insulin therapy, malignancies, other diseases of the respiratory system, other diseases of the digestive system, other diseases of the genitourinary system, gout, autoimmune disease, other diseases of the circulatory system, symptoms, signs and ill-defined conditions, diseases of the skin and subcutaneous tissues, arrhythmia, inflammatory bowel diseases, other mental disorders, heart failure, glaucoma and chronic kidney disease
Li (2021) (54)	18-59 years	2021-05-18 to 2021-06-20	China	Inactivated virus vaccines	Delta	MAI & severe	Non-clinical	Age and sex
Skowronski (2022) (55)	≥18 years	2021-05-30to2021-11-27	Canada	mRNA & Adenovirus vector vaccines	Delta & alpha & gamma	MAI & severe	Non-clinical	Age group (18–49/50–69/70–79/?80 years), sex, epi-week and region
Tang (2021) (56)	all ages	2020-12-21 to 2021-09-07	Qatar	mRNA	Beta & delta	MAI & severe	Non-clinical	Prior MAI and healthcare worker status
Thiruven-gadam (2022) (57)	all ages	2021-04-01 to 2021-05-31	India	Adenovirus vector vaccines	Delta	MAI & severe	Non-clinical	Differences in age, sex, and risk of exposure to COVID-19-positive individual

Supplementary Table 1: Summary of 66 studies included in the systematic review and meta-analysis. Methods described in (1).

**Author (year)**	**Age group**	**Study period**	**Location**	**Vaccine type**	**Circulating viruses**	**Endpoint type**	**Recruitment criteria**	**Adjustments in analysis**
Tseng (2022) (58)	≥18 years	2021-12-06 to 2021-12-31	US	mRNA	Delta & omicron	MAI & severe	Non-clinical	History of SARS-CoV-2 molecular test, preventive care, number of outpatient and virtual visits, Charlson comorbidity score, obesity, frailty index, specimen type, immunocompromised status and history of COVID-19
Winkelman (2022) (59)	≥19 years	2021-08-29 to 2021-10-30	US	mRNA & Adenovirus vector vaccines	Delta	MAI & severe	Non-clinical	NA
Collie (2022) (60)	all ages	2021-09-01 to 2021-12-07	South Africa	mRNA	Delta & omicron	Severe	Non-clinical	Age, sex, previous COVID-19 MAI, surveillance week, geographic location, and the number of CDC risk factors
Lewis (2021) (61)	≥18 years	2021-03-11 to 2021-08-15	US	mRNA	Mix	Severe	Non-clinical	Date of admission, age, sex, self-reported race and ethnicity, burden of underlying conditions, and US Health and Human Services region of the admitting hospital
Lewis2 (2022) (62)	≥18 years	2021-03-11to2021-12-15	US	Adenovirus vector vaccines	Alpha & delta	Severe	Non-clinical	Admission date (biweekly intervals), geographic region, age group, sex, and self-reported race and Hispanic ethnicity
Olson1 (2021) (63)	12-18 years	2021-06-01 to 2021-09-30	US	mRNA	Delta	Severe	Non-clinical	U.S. Census region, calendar month of admission, continuous age in years, sex, race or ethnicity
Olson2 (2022) (64)	12-18 years	2021-07-01 to 2021-10-25	US	mRNA	Delta	Severe	Non-clinical	U.S. Census region, calendar date of admission, age, sex, and race or ethnic group
Zambrano (2022) (65)	12-18 years	2021-07-01 to 2021-12-09	US	mRNA	Delta	Severe	Non-clinical	U.S. Census region, age, sex, and race and ethnicity
Altarawneh (2022) (66)	all ages	2021-12-23 to 2022-02-21	Qatar	mRNA	Omicron	Symptomatic	Non-clinical	Sex, 10-year age group, nationality, and calendar week of PCR test
Chemaitelly3 (2022) (67)	all ages	2021-12-23 to 2022-02-28	Qatar	mRNA	Omicron	Symptomatic	Non-clinical	Sex, 10-year-age group, nationality, and calendar week of PCR test

Supplementary Table 2: Summary of 66 studies by use of a clinical case definition

**Enrolment criteria**	**Studies**
Clinical case definition used	(2-42)
No clinical case definition used	(43-67)

References

1. Tsang TK, Sullivan SG, Huang X, Wang C, Wang Y, Nealon J, et al. Prior infections and effectiveness of SARS-CoV-2 vaccine in test-negative study: A systematic review and meta-analysis. medRxiv. 2022:2022.11.03.22281925.
2. Amirthalingam G, Bernal JL, Andrews NJ, Whitaker H, Gower C, Stowe J, et al. Serological responses and vaccine effectiveness for extended COVID-19 vaccine schedules in England. Nat Commun. 2021;12(1):7217.
3. Kim SS, Chung JR, Belongia EA, McLean HQ, King JP, Nowalk MP, et al. Messenger RNA Vaccine Effectiveness Against Coronavirus Disease 2019 Among Symptomatic Outpatients Aged >/=16 Years in the United States, February-May 2021. J Infect Dis. 2021;224(10):1694-8.
4. Maeda H, Saito N, Igarashi A, Ishida M, Suami K, Yagiuchi A, et al. Effectiveness of mRNA COVID-19 vaccines against symptomatic SARS-CoV-2 infections during the Delta variant epidemic in Japan: Vaccine Effectiveness Real-time Surveillance for SARS-CoV-2 (VERSUS). Clin Infect Dis. 2022;23.
5. Mallow C, Ferreira T, Shukla B, Warde P, Sosa MA, Parekh DJ, et al. Real world SARS-COV-2 vaccine effectiveness in a Miami academic institution. Am J Emerg Med. 2022;54:97-101.
6. Ranzani OT, Silva AAB, Peres IT, Antunes BBP, Gonzaga-da-Silva TW, Soranz DR, et al. Vaccine effectiveness of ChAdOx1 nCoV-19 against COVID-19 in a socially vulnerable community in Rio de Janeiro, Brazil: a test-negative design study. Clin Microbiol Infect. 2022;28(5):736 e1- e4.
7. Sheikh A, McMenamin J, Taylor B, Robertson C, Public Health S, the EIIC. SARS-CoV-2 Delta VOC in Scotland: demographics, risk of hospital admission, and vaccine effectiveness. Lancet. 2021;397(10293):2461-2.
8. Tabak YP, Sun X, Brennan TA, Chaguturu SK. Incidence and Estimated Vaccine Effectiveness Against Symptomatic SARS-CoV-2 Infection Among Persons Tested in US Retail Locations, May 1 to August 7, 2021. JAMA Netw Open. 2021;4(12):e2143346.
9. Andrews N, Tessier E, Stowe J, Gower C, Kirsebom F, Simmons R, et al. Duration of Protection against Mild and Severe Disease by Covid-19 Vaccines. N Engl J Med. 2022;386(4):340-50.
10. Cerqueira-Silva T, Katikireddi SV, de Araujo Oliveira V, Flores-Ortiz R, Junior JB, Paixao ES, et al. Vaccine effectiveness of heterologous CoronaVac plus BNT162b2 in Brazil. Nat Med. 2022;28(4):838-43.
11. Drawz PE, DeSilva M, Bodurtha P, Vazquez Benitez G, Murray A, Chamberlain AM, et al. Effectiveness of BNT162b2 and mRNA-1273 Second Doses and Boosters for Severe Acute Respiratory Syndrome Coronavirus 2 (SARS-CoV-2) Infection and SARS-CoV-2-Related Hospitalizations: A Statewide Report From the Minnesota Electronic Health Record Consortium. Clin Infect Dis. 2022;75(5):890-2.
12. Hitchings MDT, Ranzani OT, Dorion M, D'Agostini TL, de Paula RC, de Paula OFP, et al. Effectiveness of ChAdOx1 vaccine in older adults during SARS-CoV-2 Gamma variant circulation in Sao Paulo. Nat Commun. 2021;12(1):6220.
13. Nadeem I, Ul Munamm SA, Ur Rasool M, Fatimah M, Abu Bakar M, Rana ZK, et al. Safety and efficacy of Sinopharm vaccine (BBIBP-CorV) in elderly population of Faisalabad district of Pakistan. Postgrad Med J. 2022.
14. Ranzani OT, Hitchings MDT, Dorion M, D'Agostini TL, de Paula RC, de Paula OFP, et al. Effectiveness of the CoronaVac vaccine in older adults during a gamma variant associated epidemic of covid-19 in Brazil: test negative case-control study. BMJ. 2021;374:n2015.
15. Rearte A, Castelli JM, Rearte R, Fuentes N, Pennini V, Pesce M, et al. Effectiveness of rAd26-rAd5, ChAdOx1 nCoV-19, and BBIBP-CorV vaccines for risk of infection with SARS-CoV-2 and death due to COVID-19 in people older than 60 years in Argentina: a test-negative, case-control, and retrospective longitudinal study. Lancet. 2022;399(10331):1254-64.

16. Thompson MG, Burgess JL, Naleway AL, Tyner H, Yoon SK, Meece J, et al. Prevention and Attenuation of Covid-19 with the BNT162b2 and mRNA-1273 Vaccines. N Engl J Med. 2021;385(4):320-9.

17. Embi PJ, Levy ME, Naleway AL, Patel P, Gaglani M, Natarajan K, et al. Effectiveness of two-dose vaccination with mRNA COVID-19 vaccines against COVID-19-associated hospitalizations among immunocompromised adults-Nine States, January-September 2021. Am J Transplant. 2022;22(1):306-14.

18. Lauring AS, Tenforde MW, Chappell JD, Gaglani M, Ginde AA, McNeal T, et al. Clinical severity of, and effectiveness of mRNA vaccines against, covid-19 from omicron, delta, and alpha SARS-CoV-2 variants in the United States: prospective observational study. BMJ. 2022;376:e069761.

19. Luong Ngyen LB, Bauer R, Lesieur Z, Galtier F, Duval X, Vanhems P, et al. Vaccine effectiveness against COVID-19 hospitalization in adults in France: A test negative case control study. Infect Dis Now. 2022;52(1):40-3.

20. Niessen FA, Knol MJ, Hahne SJM, group Vs, Bonten MJM, Bruijning-Verhagen P. Vaccine effectiveness against COVID-19 related hospital admission in the Netherlands: A test-negative case-control study. Vaccine. 2022;40(34):5044-9.

21. Price AM, Olson SM, Newhams MM, Halasa NB, Boom JA, Sahni LC, et al. BNT162b2 Protection against the Omicron Variant in Children and Adolescents. N Engl J Med. 2022;386(20):1899-909.

22. Self WH, Tenforde MW, Rhoads JP, Gaglani M, Ginde AA, Douin DJ, et al. Comparative Effectiveness of Moderna, Pfizer-BioNTech, and Janssen (Johnson & Johnson) Vaccines in Preventing COVID-19 Hospitalizations Among Adults Without Immunocompromising Conditions - United States, March-August 2021. MMWR Morb Mortal Wkly Rep. 2021;70(38):1337-43.

23. Tartof SY, Slezak JM, Puzniak L, Hong V, Xie F, Ackerson BK, et al. Durability of BNT162b2 vaccine against hospital and emergency department admissions due to the omicron and delta variants in a large health system in the USA: a test-negative case-control study. Lancet Respir Med. 2022;10(7):689-99.

24. Tenforde MW, Olson SM, Self WH, Talbot HK, Lindsell CJ, Steingrub JS, et al. Effectiveness of Pfizer-BioNTech and Moderna Vaccines Against COVID-19 Among Hospitalized Adults Aged >/=65 Years - United States, January-March 2021. MMWR Morb Mortal Wkly Rep. 2021;70(18):674-9.

25. Tenforde MW, Patel MM, Ginde AA, Douin DJ, Talbot HK, Casey JD, et al. Effectiveness of Severe Acute Respiratory Syndrome Coronavirus 2 Messenger RNA Vaccines for Preventing Coronavirus Disease 2019 Hospitalizations in the United States. Clin Infect Dis. 2022;74(9):1515-24.

26. Tenforde MW, Self WH, Zhu Y, Naioti EA, Gaglani M, Ginde AA, et al. Protection of Messenger RNA Vaccines Against Hospitalized Coronavirus Disease 2019 in Adults Over the First Year Following Authorization in the United States. Clin Infect Dis. 2023;76(3):e460-e8.

27. Andrews N, Stowe J, Kirsebom F, Toffa S, Rickeard T, Gallagher E, et al. Covid-19 Vaccine Effectiveness against the Omicron (B.1.1.529) Variant. N Engl J Med. 2022;386(16):1532-46.

28. Lopez Bernal J, Andrews N, Gower C, Gallagher E, Simmons R, Thelwall S, et al. Effectiveness of Covid-19 Vaccines against the B.1.617.2 (Delta) Variant. N Engl J Med. 2021;385(7):585-94.

29. Lopez Bernal J, Andrews N, Gower C, Robertson C, Stowe J, Tessier E, et al. Effectiveness of the Pfizer-BioNTech and Oxford-AstraZeneca vaccines on covid-19 related symptoms, hospital admissions, and mortality in older adults in England: test negative case-control study. BMJ. 2021;373:n1088.

30. Britton A, Fleming-Dutra KE, Shang N, Smith ZR, Dorji T, Derado G, et al. Association of COVID-19 Vaccination With Symptomatic SARS-CoV-2 Infection by Time Since Vaccination and Delta Variant Predominance. JAMA. 2022;327(11):1032-41.

31. Chung JR, Kim SS, Belongia EA, McLean HQ, King JP, Nowalk MP, et al. Vaccine effectiveness against COVID-19 among symptomatic persons aged >/=12 years with reported contact with COVID-19 cases, February-September 2021. Influenza Other Respir Viruses. 2022;16(4):673-9.

32. Fleming-Dutra KE, Britton A, Shang N, Derado G, Link-Gelles R, Accorsi EK, et al. Association of Prior BNT162b2 COVID-19 Vaccination With Symptomatic SARS-CoV-2 Infection in Children and Adolescents During Omicron Predominance. JAMA. 2022;327(22):2210-9.

33. Powell AA, Kirsebom F, Stowe J, McOwat K, Saliba V, Ramsay ME, et al. Effectiveness of BNT162b2 against COVID-19 in adolescents. Lancet Infect Dis. 2022;22(5):581-3.

34. Suarez Castillo M, Khaoua H, Courtejoie N. Vaccine-induced and naturally-acquired protection against Omicron and Delta symptomatic infection and severe COVID-19 outcomes, France, December 2021 to January 2022. Euro Surveill. 2022;27(16).

35. Whitaker HJ, Tsang RSM, Byford R, Andrews NJ, Sherlock J, Sebastian Pillai P, et al. Pfizer-BioNTech and Oxford AstraZeneca COVID-19 vaccine effectiveness and immune response amongst individuals in clinical risk groups. J Infect. 2022;84(5):675-83.

36. Andrews N, Stowe J, Kirsebom F, Toffa S, Sachdeva R, Gower C, et al. Effectiveness of COVID-19 booster vaccines against COVID-19-related symptoms, hospitalization and death in England. Nat Med. 2022;28(4):831-7.

37. Chung H, He S, Nasreen S, Sundaram ME, Buchan SA, Wilson SE, et al. Effectiveness of BNT162b2 and mRNA-1273 covid-19 vaccines against symptomatic SARS-CoV-2 infection and severe covid-19 outcomes in Ontario, Canada: test negative design study. BMJ. 2021;374:n1943.

38. Ferdinands JM, Rao S, Dixon BE, Mitchell PK, DeSilva MB, Irving SA, et al. Waning 2-Dose and 3-Dose Effectiveness of mRNA Vaccines Against COVID-19-Associated Emergency Department and Urgent Care Encounters and Hospitalizations Among Adults During Periods of Delta and Omicron Variant Predominance - VISION Network, 10 States, August 2021-January 2022. MMWR Morb Mortal Wkly Rep. 2022;71(7):255-63.

39. Grannis SJ, Rowley EA, Ong TC, Stenehjem E, Klein NP, DeSilva MB, et al. Interim Estimates of COVID-19 Vaccine Effectiveness Against COVID-19-Associated Emergency Department or Urgent Care Clinic Encounters and Hospitalizations Among Adults During SARS-CoV-2 B.1.617.2 (Delta) Variant Predominance - Nine States, June-August 2021. MMWR Morb Mortal Wkly Rep. 2021;70(37):1291-3.

40. Kirsebom FCM, Andrews N, Stowe J, Toffa S, Sachdeva R, Gallagher E, et al. COVID-19 vaccine effectiveness against the omicron (BA.2) variant in England. Lancet Infect Dis. 2022;22(7):931-3.

41. Nasreen S, Chung H, He S, Brown KA, Gubbay JB, Buchan SA, et al. Effectiveness of COVID-19 vaccines against symptomatic SARS-CoV-2 infection and severe outcomes with variants of concern in Ontario. Nat Microbiol. 2022;7(3):379-85.

42. Suarez Castillo M, Khaoua H, Courtejoie N. Vaccine effectiveness and duration of protection against symptomatic infections and severe Covid-19 outcomes in adults aged 50 years and over, France, January to mid-December 2021. Glob Epidemiol. 2022;4:100076.

43. Bruxvoort KJ, Sy LS, Qian L, Ackerson BK, Luo Y, Lee GS, et al. Effectiveness of mRNA-1273 against delta, mu, and other emerging variants of SARS-CoV-2: test negative case-control study. BMJ. 2021;375:e068848.

44. Butt AA, Omer SB, Yan P, Shaikh OS, Mayr FB. SARS-CoV-2 Vaccine Effectiveness in a High-Risk National Population in a Real-World Setting. Ann Intern Med. 2021;174(10):1404-8.

45. Husin M, Tok PSK, Suah JL, Thevananthan T, Tng BH, Peariasamy KM, et al. Real-world effectiveness of BNT162b2 vaccine against SARS-CoV-2 infection among adolescents (12 to 17-year-olds) in Malaysia. Int J Infect Dis. 2022;121:55-7.

46. Pardo-Seco J, Mallah N, Lopez-Perez LR, Gonzalez-Perez JM, Roson B, Otero-Barros MT, et al. Evaluation of BNT162b2 Vaccine Effectiveness in Galicia, Northwest Spain. Int J Environ Res Public Health. 2022;19(7).

47. Sritipsukho P, Khawcharoenporn T, Siribumrungwong B, Damronglerd P, Suwantarat N, Satdhabudha A, et al. Comparing real-life effectiveness of various COVID-19 vaccine regimens during the delta variant-dominant pandemic: a test-negative case-control study. Emerg Microbes Infect. 2022;11(1):585-92.

48. Abu-Raddad LJ, Chemaitelly H, Bertollini R, National Study Group for C-V. Waning mRNA-1273 Vaccine Effectiveness against SARS-CoV-2 Infection in Qatar. N Engl J Med. 2022;386(11):1091-3.

49. Abu-Raddad LJ, Chemaitelly H, Butt AA, National Study Group for C-V. Effectiveness of the BNT162b2 Covid-19 Vaccine against the B.1.1.7 and B.1.351 Variants. N Engl J Med. 2021;385(2):187-9.

50. Andrejko KL, Pry J, Myers JF, Jewell NP, Openshaw J, Watt J, et al. Prevention of Coronavirus Disease 2019 (COVID-19) by mRNA-Based Vaccines Within the General Population of California. Clin Infect Dis. 2022;74(8):1382-9.

51. Chemaitelly H, Tang P, Hasan MR, AlMukdad S, Yassine HM, Benslimane FM, et al. Waning of BNT162b2 Vaccine Protection against SARS-CoV-2 Infection in Qatar. N Engl J Med. 2021;385(24):e83.

52. Chemaitelly H, Yassine HM, Benslimane FM, Al Khatib HA, Tang P, Hasan MR, et al. mRNA-1273 COVID-19 vaccine effectiveness against the B.1.1.7 and B.1.351 variants and severe COVID-19 disease in Qatar. Nat Med. 2021;27(9):1614-21.

53. Corrao G, Franchi M, Rea F, Cereda D, Barone A, Borriello CR, et al. Protective action of natural and induced immunization against the occurrence of delta or alpha variants of SARS-CoV-2 infection: a test-negative case-control study. BMC Med. 2022;20(1):52.

54. Li XN, Huang Y, Wang W, Jing QL, Zhang CH, Qin PZ, et al. Effectiveness of inactivated SARS-CoV-2 vaccines against the Delta variant infection in Guangzhou: a test-negative case-control real-world study. Emerg Microbes Infect. 2021;10(1):1751-9.

55. Skowronski DM, Febriani Y, Ouakki M, Setayeshgar S, El Adam S, Zou M, et al. Two-Dose Severe Acute Respiratory Syndrome Coronavirus 2 Vaccine Effectiveness With Mixed Schedules and Extended Dosing Intervals: Test-Negative Design Studies From British Columbia and Quebec, Canada. Clin Infect Dis. 2022;75(11):1980-92.

56. Tang P, Hasan MR, Chemaitelly H, Yassine HM, Benslimane FM, Al Khatib HA, et al. BNT162b2 and mRNA-1273 COVID-19 vaccine effectiveness against the SARS-CoV-2 Delta variant in Qatar. Nat Med. 2021;27(12):2136-43.

57. Thiruvengadam R, Awasthi A, Medigeshi G, Bhattacharya S, Mani S, Sivasubbu S, et al. Effectiveness of ChAdOx1 nCoV-19 vaccine against SARS-CoV-2 infection during the delta (B.1.617.2) variant surge in India: a test-negative, case-control study and a mechanistic study of post-vaccination immune responses. Lancet Infect Dis. 2022;22(4):473-82.

58. Tseng HF, Ackerson BK, Luo Y, Sy LS, Talarico CA, Tian Y, et al. Effectiveness of mRNA-1273 against SARS-CoV-2 Omicron and Delta variants. Nat Med. 2022;28(5):1063-71.

59. Winkelman TNA, Rai NK, Bodurtha PJ, Chamberlain AM, DeSilva M, Jeruzal J, et al. Trends in COVID-19 Vaccine Administration and Effectiveness Through October 2021. JAMA Netw Open. 2022;5(3):e225018.

60. Collie S, Champion J, Moultrie H, Bekker LG, Gray G. Effectiveness of BNT162b2 Vaccine against Omicron Variant in South Africa. N Engl J Med. 2022;386(5):494-6.

61. Lewis NM, Naioti EA, Self WH, Ginde AA, Douin DJ, Keipp Talbot H, et al. Effectiveness of mRNA Vaccines Against COVID-19 Hospitalization by Age and Chronic Medical Conditions Burden Among Immunocompetent US Adults, March-August 2021. J Infect Dis. 2022;225(10):1694-700.

62. Lewis NM, Self WH, Gaglani M, Ginde AA, Douin DJ, Keipp Talbot H, et al. Effectiveness of the Ad26.COV2.S (Johnson & Johnson) Coronavirus Disease 2019 (COVID-19) Vaccine for Preventing COVID-19 Hospitalizations and Progression to High Disease Severity in the United States. Clin Infect Dis. 2022;75(Suppl 2):S159-S66.

63. Olson SM, Newhams MM, Halasa NB, Price AM, Boom JA, Sahni LC, et al. Effectiveness of Pfizer-BioNTech mRNA Vaccination Against COVID-19 Hospitalization Among Persons Aged 12-18 Years - United States, June-September 2021. MMWR Morb Mortal Wkly Rep. 2021;70(42):1483-8.

64. Olson SM, Newhams MM, Halasa NB, Price AM, Boom JA, Sahni LC, et al. Effectiveness of BNT162b2 Vaccine against Critical Covid-19 in Adolescents. N Engl J Med. 2022;386(8):713-23.

65. Zambrano LD, Newhams MM, Olson SM, Halasa NB, Price AM, Boom JA, et al. Effectiveness of BNT162b2 (Pfizer-BioNTech) mRNA Vaccination Against Multisystem Inflammatory Syndrome in Children Among Persons Aged 12-18 Years - United States, July-December 2021. MMWR Morb Mortal Wkly Rep. 2022;71(2):52-8.

66. Altarawneh HN, Chemaitelly H, Ayoub HH, Tang P, Hasan MR, Yassine HM, et al. Effects of Previous Infection and Vaccination on Symptomatic Omicron Infections. N Engl J Med. 2022;387(1):21-34.

67. Chemaitelly H, Ayoub HH, AlMukdad S, Coyle P, Tang P, Yassine HM, et al. Duration of mRNA vaccine protection against SARS-CoV-2 Omicron BA.1 and BA.2 subvariants in Qatar. Nat Commun. 2022;13(1):3082.
